# Clinical characteristics and management outcomes of acute retinal necrosis

**DOI:** 10.1038/s41598-023-44310-4

**Published:** 2023-10-07

**Authors:** Elham Shiezadeh, Seyedeh Maryam Hosseini, Elham Bakhtiari, Alireza Mojarrad, Mehrdad Motamed Shariati

**Affiliations:** Eye Research Center, Khatam Al-Anbia Eye Hospital, Mashhad University of Medical Sciences, Gharani Boulevard, Mashhad, Iran

**Keywords:** Diseases, Health care

## Abstract

This study investigates patient’s clinical characteristics and management outcomes of PCR-positive Acute Retinal Necrosis (ARN). The patient’s clinical characteristics of the disease, and therapeutic approaches were assessed. Data from the medical records of 40 eyes of 40 patients were analyzed. The mean ± standard deviation (SD) of the age of the patients was 47.8 ± 14.1 years (16–84 years old). The median follow-up time was 160 days, with a range of 120–370 days. The mean ± SD of patients' primary and final BCVA was 1.24 ± 0.78 and 1.08 ± 0.86 LogMAR, respectively. The final BCVA increased significantly after the treatment in the last follow-up period in patients who did not undergo PPV (*p* = 0.029). Although, vision changes were not statistically significant in patients who underwent PPV (*p* = 0.549). 75% of our patients had a positive aqueous PCR for VZV, and the second most common causative agents were CMV and HSV (10% for each). Besides, rhegmatogenous retinal detachment (RRD) occurred in 25% of our patients. Our analysis showed that the presenting visual acuity and RRD occurrence are the significant prognostic factors for final blindness in ARN.

## Introduction

Acute retinal necrosis (ARN) is an infectious pan-uveitis syndrome caused mainly by the herpes viruses family, including herpes simplex virus (HSV-1, HSV-2) and varicella-zoster virus (VZV) with some distinct clinical features^1^. Most patients are immunocompetent, however, the disease shows more aggression in immunocompromised subjects^2^. ARN presents with circumferential progressive necrotizing retinitis accompanying vitreous inflammation. Severe blinding complications associated with ARN include retinal detachment (RD) with proliferative vitreoretinopathy (PVR), optic neuritis, macular involvement, and ischemic vasculitis^3,4^. Prompt antiviral treatment is necessary to manage inflammation and prevent devastating complications and the involvement of the fellow eye^2^. The consensus treatment includes the induction phase with intravenous acyclovir or oral valacyclovir and the maintenance phase. Intravitreal injection of antivirals, such as foscarnet or ganciclovir, is considered an adjuvant treatment. Several studies have shown the beneficial effects of antiviral intravitreal injections in reducing the incidence of RD and improving visual outcomes^5,6^. Although administering systemic corticosteroids is controversial, based on the results of some studies, it is recommended to subside the host immunoinflammatory response^7–9^. However, the protective effects of antiviral intravitreal injections and systemic corticosteroids on final vision or complications such as RD have not yet been proven.

Despite applying various systemic and local treatments for ARN, RD and permanent vision loss are the therapeutic challenges^10^. The exact role of pars plana vitrectomy in preventing complications such as RD is unclear. Some previous studies showed that early vitrectomy might decrease the risk of RD, especially in patients whose physicians cannot perform barrier lasers^11^. Besides, the importance of multiple intravitreal injections of antivirals in the final vision and prognosis has not yet been clarified.

This study aims to evaluate ARN patients' demographic and clinical characteristics. Furthermore, we will report and discuss the diverse therapeutic efforts, visual outcomes, and complications.

## Results

### Demographics and baseline clinical characteristics

In this study, data from the medical records of 40 patients diagnosed with ARN between 2015 and 2022 were analyzed. Of these, 26 (65%) were men, and 14 (35%) were women. All patients had a positive aqueous polymerase chain reaction (PCR) result for the causative agent. The mean ± SD of the age of the patients was 47.8 ± 14.1 years with a range of 16–84 years old. Twenty-four cases (64%) had right eye involvement. The highest number of the disease presents in the spring season, with 14 patients (35%), followed by autumn, winter, and summer. Regarding the season of the disease onset, we found no statistically significant difference. The time interval between the onset of symptoms and the patient’s examination was one to nine days (median: 2 days). The standard treatment protocol, discussed in the methods section, was administered to all patients. Systemic antiviral treatment was not discontinued in any case due to drug toxicity. The median number and duration of hospitalization were 2 and 7 days, respectively.

Demographics and clinical characteristics of ARN patients at presentation are summarized in Table 1. Considering the extent of retinitis, most patients (70%) had less than three quadrants of involvement at the time of the disease onset. While 70% of patients showed moderate or higher anterior segment inflammation (3 + or 4 + based on SUN Working Group), 80% had moderate or lower grades of vitreous haziness (0 to 2 + based on SUN Working Group). Furthermore, we found a non-granulomatous inflammation in 77.5% of patients. The most common positive PCR result was VZV (75%). Two (5%) and eight patients (20%) had RRD at the time of presentation and in the follow-up period, respectively. Late RRD has occurred with a median of 66 days (Range: 17–130 days) after the initial presentation of the disease.

**Table 1:** Demographic and clinical characteristics of ARN patients at presentation.

Age (mean ± SD)		47.8 ± 14.1
Gender N (%)		
	Male	26 (65)
	Female	14 (35)
Laterality N (%)		
	Right eye	24 (64)
	Left eye	16 (36)
Season N (%)		
	Spring	14 (35)
	Summer	7 (17.5)
	Autumn	12 (30)
	Winter	7 (17.5)
The extent of retinitis (number of involved quadrants) N (%)		
	1 Q	10 (25)
	2 Q	18 (45)
	3 Q	9 (22.5)
	4 Q	3 (7.5)
Baseline visual acuity (LogMAR), mean (Range)		1 (0.5–1.85)
Type of inflammation N		
(%)	Granulomatous	9 (22.5)
	Non-granulomatous	31 (77.5)
AC cells, N (%)		
	0	1 (2.5)
	+ 1	5 (12.5)
	+ 2	6 (15)
	+ 3	16 (40)
	+ 4	12 (30)
Vitreous haziness, N (%)		
	0	3 (7.5)
	+ 1	16 (40)
	+ 2	13 (32.5)
	+ 3	5 (12.5)
	+ 4	3 (7.5)
PCR results N (%)		
	VZV	30 (75)
	EBV	1 (2.5)
	Covid-19	1 (2.5)
	HSV	4 (10)
	CMV	4 (10)
RRD, N (%)	At presentation	2 (5)
	In the follow-up period	8 (20)
	No RRD	30 (75)

### Therapeutic procedures and complications of ARN

The median follow-up time was 160 days, ranging from 120to 370 days. Thirty-six patients (90%) underwent intravitreal ganciclovir injection for at least one time. A total of 17 patients underwent PPV, of which 4 had early PPV. The indication of PPV was RRD in 10 patients (2 patients in the early and 8 patients in the late PPV groups) and severe dense vitritis or vitreous membranes with severe retinal traction in 7 patients (2 patients in the early and 5 patients in the late PPV groups). Silicone oil was the most used tamponade in PPV (70.5%), and all patients underwent endolaser photocoagulation (ELP) during the surgery. We found no statistically significant difference in the final visual outcome between the two categories of patients who underwent PPV (RRD versus no-RRD patients) (*p* = 0.193). Also, there was no statistically significant difference in the final visual outcome between the two groups of early and late vitrectomy (*p* = 0.34). A prophylactic barrier laser was performed in 17 patients (42.5%). The most observed complication in the patients was cataract (35%), followed by the RRD (20%), and epiretinal membrane (ERM) (12.5%) (Table 2). Hypotonia, macular hole (MH), and exudative RD were reported in one patient each.

**Table 2:** Therapeutic procedures and complications during the follow-up period.

Follow-up duration time (days), median (Range)		160 (12–370)
Intravitreal ganciclovir injections, N (%)		
	No injection	4 (10)
	1–2 injections	13 (32.5)
	More than two injections	23 (57.5)
Pars plana vitrectomy, N		
(%)	No	23 (57.5)
	Early	4 (10)
	Late	13 (32.5)
Tamponade, N (%)		
	No tamponade	4 (23.5)
	Silicone oil	12 (70.5)
	Gas	1 (6)
Endolaser photocoagulation, N (%)		
	Yes	17 (100)
	No	0 (0)
Prophylactic barrier laser, N (%)		17 (42.5)
Complications during the follow-up period, N (%)		
	CME	4 (10)
	ERM	5 (12.5)
	Glaucoma	2 (5)
	Cataract	14 (35)
	Late RRD	8 (20)

The time-interval between the disease presentation and the first intravitreal ganciclovir injection was 0 to 4 days as follows: 30 patients underwent intravitreal injection 24 h after the first presentation to us, 4 patients were injected in the second day, one patient was injected in the third and one was injected in the fourth day of the disease presentation. The time of the next injection depends on the clinical condition of the patient, the surgeon’s discretion, and the patient’s preference for performing an invasive treatment. Considering the number of intravitreal ganciclovir injections, we subdivided the patients into three groups: patients with more than two times of injections, patients with one or two times of injections, and patients with no injections. The results of the ANOVA test and post-hoc analysis showed that patients who underwent ganciclovir intravitreal injections more than two times received a significantly longer duration of systemic antiviral treatment (*p* = 0.006). We found no statistically significant difference in the incidence of RRD, final visual acuity, extent of retinitis, and anterior segment inflammation in the three sub-groups of patients regarding the intravitreal ganciclovir injections.

Comparing the incidence of complications, including cataracts, RRD, ERM, CME, and glaucoma, in different groups of patients in terms of the extent of retinitis involvement (number of involved quadrants) with the Pearson Chi-square test showed that there is no significant difference in the incidence of complications between these groups.

The multivariate regression analysis was established to investigate the predictive value of the possible variables, including vitreous and anterior chamber inflammation grade, prophylactic BL, primary visual acuity, the extent of retinitis, and the number of ganciclovir injections on the occurrence of RRD. In this model, we found no statistically significant variable.

We investigated the number of intravitreal ganciclovir injections in different groups of patients regarding the PPV surgery (No PPV, early PPV, and late PPV) with the Pearson Chi-square test. The results showed no statistically significant difference (*p* = 0.231) in the number of intravitreal ganciclovir injections between these groups. Besides, the results of the Pearson Chi-square test showed no statistically significant difference in the extent of retinitis (the number of involved quadrants), and the anterior segment inflammation grade between the three groups of patients regarding the PPV surgery (No PPV, early PPV, and late PPV) (*p* = 0.737, and 0.538, respectively).

### Visual outcomes

The mean ± standard deviation (sd) of the primary and final BCVA of patients was 1.24 ± 0.78 LogMAR and 1.08 ± 0.86 LogMAR, respectively; which was significantly worse in the vitrectomy group than patients with no surgical treatment (*p* = 0.05, 0.04, respectively). Also, the median (interquartile range) of the primary and final BCVA of patients were 1 (0.5, 1.85) and 1 (0.3, 1.85), respectively. The Wilcoxon Signed Ranks test showed that the final visual acuity increased significantly after the treatment in the last follow-up period in patients who did not undergo PPV (*p* = 0.027). Although, changes in visual acuity were not statistically significant in patients who underwent early or late PPV (*p* = 0.109, *p* = 0.09, respectively) (Table 3).

**Table 3:** VA changes in patients from the presentation to the last follow-up visit.

		Number of patients	Baseline VA (LogMAR), Median (IQR)	Last follow-up VA (LogMAR), Median (IQR)	*P*-Value*
Total patients		40	1 (0.5, 1.85)	1 (0.3, 1.85)	0.093
	No vitrectomy	23	1 (0.5, 1.85)	0.7 (0.3, 1.85)	0.027
	Early vitrectomy	4	0.5 (1.87, 1.90)	1.85 (0.61, 2.18)	0.109
	Late vitrectomy	13	1 (0.5, 1.85)	1 (0.4, 1.85)	0.090

*Wilcoxon Signed Ranks test. IQR: Inter-quartile range.

We used the logit model to investigate if the final vision loss could be predicted with some clinically critical independent variables, including the extent of retinitis, number of ganciclovir injections, RRD, and primary visual acuity. All patients were divided into two groups legally blind (LogMAR ≥ 1) and not blind (LogMAR < 1) based on their final VA. Initial VA and RRD were significantly different in the two groups of patients regarding the final visual acuity (Table 4).

**Table 4:** Comparison of clinical characteristics associated with final visual outcomes.

		Final visual acuity		
		≥ 20/200	< 20/200	*P*-Value
Age		46.75	50.5	0.445
Initial visual acuity (LogMAR)		0.95	1.7	**0.002**^**a**^
Vitreous inflammation				0.972
	0	2	1	
	+ 1	8	8	
	+ 2	9	4	
	+ 3	3	2	
	+ 4	2	1	
Anterior chamber inflammation				0.830
	0	1	0	
	+ 1	2	3	
	+ 2	4	2	
	+ 3	11	5	
	+ 4	6	6	
Extent of retinitis				0.298
	1Q	7	3	
	≥ 2Q	17	13	
RRD				**0.029**^**b**^
		3	7	

Values are expressed as mean ± standard deviation, except where indicated as the number of subjects. A *P*-Value in bold indicates statistical significance (*P* < 0.05).

^a^Derived from an independent *t*-test.

^b^Derived from a chi-square test.

Significant values are in [bold].

Possible prognostic factors were input in the logistic regression model for the risk of final vision loss. The results of the logistic regression analysis are summarized in Table 5. In this model, RRD (OR, 0.26; 95% CI, 0.01–0.655; *p* = 0.027) and the primary VA (OR, 7.18; 95% CI, 1.42–16.82; *p* = 0.009) revealed statistical significance for final blindness.

**Table 5:** Logistic regression analysis for 40 eyes with ARN: estimation of risk factors associated with final vision loss (LogMAR ≥ 1).

Variable	Odds ratio	95% confidence interval	*P*-Value
Extent of retinitis	4.14	0.369–46.46	0.084
Gancyclovir injection	1.11	0.185–6.76	0.404
RRD	0.26	0.01–0.655	**0.027**
Primary visual acuity	7.18	1.42–16.82	**0.009**
PPV	11.03	0.501–242.98	0.128

Significant values are in [bold].

## Discussion

In this study, we investigated the clinical characteristics, management, and final visual outcomes of PCR-positive ARN patients. Almost two-thirds of our patients were male. However, the results of previous studies showed no sexual preference regarding the prevalence of ARN^12^. In terms of seasonal prevalence, our results were unremarkable. Hedayatfar et al., in a study in 2020, showed significant seasonal variability in the incidence of ARN, with the highest incidence during the first half of the year^13^. Previous studies have reported a considerable variation in the severity of inflammation at presentation^14^, possibly due to the time interval between the onset of symptoms and the patient’s examination^15,16^. Increasing the time interval between the onset of symptoms and ocular examinations can lead to a worsening of vision prognosis. Due to the progressive nature of retinitis, delayed antiviral treatment can lead to increasing the extent of retinitis and deterioration of vision^8^. In our study, 30% of patients had 3 or 4 retinal quadrants involvement, and 80% had moderate or lower vitreous haziness. However, 70% of our patients presented with moderate and higher anterior chamber inflammation.

The most causative viral agent for ARN is VZV, estimated positive in 50%-70% of cases, followed by HSV^17^. While 75% of our patients had a positive aqueous PCR for VZV, the second most common causative agents were CMV and HSV (10% for each). An important point is the relatively high prevalence of positive PCR test results for CMV in ARN patients in this cohort. CMV has been reported rarely as the causative organism for ARN^18^. Ganatra et al. in a study showed the prevalence of approximately 3% for CMV as the causative organism in a case series of ARN patients^19^. Due to the lack of sufficient information about the causes of ARN in this region, we cannot attribute it to the regional variation of causes. It seems that this observation is just coincidental.

In the present study, cataracts, RRD, and ERM formation were the most common complications in ARN. RRD has been observed in 25% of our patients, which is lower than the results of previous studies, with an estimated prevalence of 30–70%^3,5,20^. The relatively low prevalence of RRD can be attributable to the short time interval from the onset of symptoms to the start of treatment, appropriate barrier laser because of less vitreous inflammation and haziness, and aggressive treatments including multiple intravitreal ganciclovir injections. However, the results of the current study could not find a significant association between the occurrence of RD and ocular risk factors including more severe inflammation of the anterior segment and vitreous, the extent of retinitis, as well as therapeutic measures including repeated intravitreal injections of antiviral and prophylactic BL. Numerous and prolonged use of intravitreal antiviral injections is suggested to reduce the incidence of RRD^21^. In this study, thirty-six patients (90%) experienced at least one, and more than half underwent three or more intravitreal ganciclovir injections. Due to the small number of patients with no intravitreal injections, we couldn’t evaluate the effect of intravitreal ganciclovir injection in the prevention of RD compared to no injection. We found no statistically significant difference in the incidence of RRD in the three sub-groups of patients regarding the intravitreal ganciclovir injections. Besides, almost 50% of our patients underwent prophylactic barrier lasers. The prophylactic effect of barrier lasers against RRD has not been proven in previous studies^5,22,23^. In a study by Choi et al., retinitis with more than three quadrants of involvement and poor primary visual acuity were the significant risk factors for RRD in ARN. They showed that intravitreal antiviral injection, shorter intervals between ARN diagnosis and treatment initiation, PPV, and intraoperative ELP had a significant association with a decreased risk of late-onset RD^11^. However, we couldn’t find any significant predictive variable for RRD occurrence in this cohort with the logistic regression model. Besides, regarding the short number of patients in this cohort, the role of early PPV in preventing RRD in patients with severe inflammation remained unclear. In this cohort, 42.5% of patients underwent PPV and ELP, while the frequency of RRD was 25%. Considering the nature of necrotizing retinitis, in severe vitreous inflammation and lack of good view to perform barrier laser early PPV along with ELP seems reasonable^24,25^. The preventive role of PPV in reducing the incidence of RD has been shown in previous studies^23,26^. However, in this study, due to the small number of patients in the early vitrectomy group, it was not possible to evaluate the preventive effect of early vitrectomy on the incidence of RRD. Of the four patients in the early vitrectomy group, 2 patients underwent surgery due to RRD. The indication of surgery for the other two patients was severe and dense vitritis and membranous hyper-echo lesions in the B-scan ultrasonography. As RRD is a major cause of blindness in ARN, prevention of its occurrence can play a significant role in improving the patient’s visual prognosis. Thus, if the preventive value of prophylactic early vitrectomy in the occurrence of RRD is proven, it seems reasonable to expect visual outcomes improvement.

Our analysis showed that the primary visual acuity and RRD occurrence are the significant prognostic factors for final blindness. However, the extent of retinitis, anterior chamber inflammation, vitreous haziness grade, number of ganciclovir injections, and barrier laser are other investigated risk factors that were not significantly different among the two groups of patients regarding the final visual acuity. Prior studies have demonstrated poor initial vision, treatment delay, macular and optic nerve head involvement, extensive retinitis, and RRD are associated with worse visual outcomes^27^. The patient’s low initial vision is mainly due to severe vitritis and macular or optic nerve involvement^22^. The necrotizing nature of the retinitis leads to permanent visual loss. In addition, the disease progresses relatively quickly in the absence of antiviral treatment. The greater the extent of retinal involvement, the greater the risk of complications such as RD and blindness^11^.

In this cohort, despite following the standard treatment protocol and multiple and prolonged uses of intravitreal ganciclovir injections, the improvement of final VA was not statistically significant in all patients and the PPV sub-group of patients. Our assessments of the changes in the VA indicate a significant improvement in the no-PPV subgroups of patients. Lower initial inflammation and more appropriate response to treatment in the no-PPV subgroups of patients can justify this difference. Also, it indicates that in this cohort, patients with more severe inflammation or complications, such as RRD, are selected for PPV. Preventive early vitrectomy has not been confirmed effective in reducing the risk of final blindness. However, the number of patients in this group was relatively low. Besides, this study has not been designed to investigate if early vitrectomy prevents RD and improves final visual outcomes. More studies with prospective design need to investigate whether early PPV effectively prevents blindness in ARN.

This study has some limitations. The retrospective nature of this cohort is the most prominent limitation. Furthermore, only patients with a positive aqueous PCR result were included in our study, while the diagnosis of ARN is based on clinical findings.

## Conclusion

In this retrospective cohort of 40 eyes with ARN, we showed that initial visual acuity and RRD are the main prognostic factors for final blindness. The incidence of RRD was 20% during the follow-up period, possibly due to appropriate barrier laser because of less vitreous inflammation and haziness, and aggressive treatments, including multiple intravitreal ganciclovir injections.

## Methods

In this retrospective study, we evaluated patients’ medical records with the diagnosis of ARN at the uveitis department of Khatam-Al-Anbia Eye Hospital, affiliated with Mashhad University of Medical Sciences (MUMS), between 2015 and 2022. Those who met the diagnostic criteria of ARN according to the American Uveitis Society and had a positive aqueous polymerase chain reaction (PCR) result for the herpes virus family were included in the study. The MUMS ethical committee accepted this study, which adhered to the Declaration of Helsinki (approval number: IR.MUMS.MEDICAL.REC.1399.427). The data were anonymized before the analysis.

### Data collection

The required data were gathered as follows: patients’ demographic characteristics, the presenting symptoms, initial best-corrected visual acuity (BCVA), clinical characteristics of the disease and intraocular inflammation grade, coexisting conditions (such as an immunosuppressed state), therapeutic approaches including intravitreal ganciclovir injections, the rate of early and late pars plana vitrectomy (PPV), prophylactic barrier laser photocoagulation, and visual outcomes at the last follow-up visit. We defined early PPV as the procedure performed in the first two weeks of disease presentation due to severe dense vitritis or RD and late PPV as the procedure performed after two weeks.

We used the logMAR scale to analyze the visual data quantitatively. In this way, we considered 1.85 logMAR as the vision of counting fingers, 2.30 as the vision of hand motion, 2.48 as the vision of light perception (LP), and 3 as the vision of no light perception (NLP). Clinical characteristics of ARN were as follows: The degree of anterior segment inflammation and vitritis (based on SUN Working Group)^28^, quadrants of the involved retina, presence of RD at presentation, and late RD (the incidence of RD after two weeks of the ARN presentation).

### Treatment protocol

All ARN patients treated with systemic antiviral (intravenous acyclovir 1500 mg/m^2^/day or oral valacyclovir 2 gr three times/day) for 7–10 days as the induction phase, followed by oral valacyclovir (1 gr three times/day) or acyclovir (800 mg five times/day) for at least three months as the maintenance phase. Adjuvant treatments include oral prednisolone 0.5–1 mg/Kg/day, and intravitreal ganciclovir (2 mg/0.1 ml) injections are considered in patients with severe vitreous inflammation, extended retinal necrosis, and optic nerve or macular involving retinitis. Surgical procedures include 3-port 23-gauge PPV and endolaser photocoagulation (ELP) in cases with severe vitritis or RD. Silicon oil tamponade was utilized in cases with RD or severe retinal necrosis.

### Statistical analysis

We used Statistical Package for Social Sciences (SPSS) software version 22 (IBM SPSS Statistics, IBM Corporation, Chicago, IL) for statistical analysis. The Shapiro–Wilk test was utilized to analyze the distribution of data. The characteristics of the subjects are evaluated by descriptive statistical methods, including central indices and indices of dispersion. We used the Wilcoxon Signed Ranks test to evaluate the changes in visual acuity from the presentation to the last follow-up visit. Besides, the Pearson Chi-square test was used to assess whether there is a significant relation between different types of complications with the inflammation grade and the number of intravitreal ganciclovir injections during the follow-up period.

### Ethics approval and consent to participate

This study is by the declaration of Helsinki and it was approved by the institutional review board and ethics committee of the Mashhad University of Medical Sciences (approval number: IR.MUMS.MEDICAL.REC.1399.427). Informed consent for participation was acquired from the patients.

## Data Availability

The data supporting this study’s findings are available from the corresponding author upon reasonable request.
